# The association between gut microbiome affecting concomitant medication and the effectiveness of immunotherapy in patients with stage IV NSCLC

**DOI:** 10.1038/s41598-021-02598-0

**Published:** 2021-12-02

**Authors:** M. V. Verschueren, C. M. Cramer - van der Welle, M. Tonn, F. M. N. H. Schramel, B. J. M. Peters, E. M. W. van de Garde

**Affiliations:** 1 Department of Clinical Pharmacy, St. Antonius Hospital, Koekoekslaan 1, 3435 CM Utrecht/Nieuwegein, The Netherlands; 2 Santeon Hospital Group, Utrecht, The Netherlands; 3 Department of Pulmonary Diseases, St. Antonius Hospital, Utrecht/Nieuwegein, The Netherlands; 4 Division of Pharmacoepidemiology and Clinical Pharmacology, Department of Pharmaceutical Sciences, Utrecht University, Utrecht, The Netherlands

**Keywords:** Epidemiology, Cancer immunotherapy

## Abstract

Several observational studies suggested that gut microbiome-affecting-medication impairs the effectiveness of immunotherapy in patients with metastatic non-small-cell lung cancer (NSCLC). We postulated that if the effectiveness of immunotherapy is affected by drug-related changes of the microbiome, a stronger association between the use of co-medication and overall survival (OS) will be observed in patients treated with immunotherapy as compared to patients treated with chemotherapy. In a retrospective matched cohort study, immunotherapy patients were matched (1:1) to patients treated with chemotherapy in the pre immunotherapy era. The association between the use of antibiotics, opioids, proton pump inhibitors, metformin and other antidiabetics on OS was assessed with multivariable cox-regression analyses. Interaction tests were applied to investigate whether the association differs between patients treated with immuno- or chemotherapy. A total of 442 patients were studied. The use of antibiotics was associated with worse OS (adjusted Hazard Ratio (aHR) 1.39, *p* = 0.02) independent of the type of therapy (chemotherapy or immunotherapy). The use of opioids was also associated with worse OS (aHR 1.33, *p* = 0.01). The other drugs studied showed no association with OS. Interaction term testing showed no effect modification by immuno- or chemotherapy for the association of antibiotics and opioids with OS. The use of antibiotics and opioids is similarly associated with worse outcomes in both chemotherapy and immunotherapy treated NSCLC patients. This suggests that the association is likely to be a consequence of confounding rather than disturbing the composition of the microbiome.

## Introduction

Lung cancer is the leading cause of cancer-related deaths worldwide. In 2012, the World Health Organisation (WHO) estimated that of the 1.6 million lung cancer related deaths, non-small-cell lung cancer (NSCLC) is the most frequent histological type, representing 85% of cases^1^.

Since the beginning of systemic treatment of stage III and IV NSCLC patients, platinum-doublet chemotherapy has been the first-choice treatment. With the development of new therapies, more effective treatment options have become available in addition to the platinum-doublet therapy^2,3^. For patients without driver mutations, immunotherapies targeting immune checkpoints, such as the PD-1/PD-L1 pathway, have become an important new treatment option. Currently, four PD-1/PD-L1 inhibitors (pembrolizumab, nivolumab, durvalumab and atezolizumab) have been approved for treatment of patients with advanced NSCLC.

The approval of immunotherapy, with or without chemotherapy, is based on several phase III studies demonstrating superior efficacy in a population meeting the strict in- and exclusion criteria^4–6^. For example, patients with present active infections, autoimmune conditions, and other comorbidities often requiring concomitant medication are not included in these trials. Therefore, the impact of these factors, including the use of concomitant medication on the efficacy of immunotherapy, cannot be studied from clinical trial data.

Recent studies demonstrate that the microbial flora plays an important role in modulation of immunotherapy effectiveness by affecting the tumor immuno-microenvironment^7^. It is well known that the microbiome can vary significantly from one individual to another, which has been proposed as an explanation of the variability of response to immunotherapy^8^. Routy et al. showed that changes in composition of the gut microbiome negatively influence the outcome of PD-1 inhibition by immunotherapy in mice and patients. Additionally, the study found that the replacement of deleterious microbial flora by a favorable one, can restore the efficacy of the immunotherapy response in mice^9^. The composition of the human microbiome is influenced by several factors such as host genetics, lifestyle factors and the use of antibiotics. Besides antibiotics, recent microbiome studies showed that many other drugs can also alter the composition of the microbiome, as investigated with high-throughput drug screens and metagenomics analyses^10,11^.

Accumulating observational studies show that antibiotic treatment associates significantly with attenuated clinical outcomes in NSCLC patients treated with immunotherapy^9,12,13^. However, the majority of these studies analyzed the effects of antibiotics on survival outcomes in cohort studies including only immunotherapy patients. This makes the results susceptible for bias due to confounding by indication, a situation in which patient characteristics—rather than the presence of concomitant medication- are independent predictors of clinical outcomes. Therefore, it is still an active area of debate whether or not there is a causal link between antibiotic related changes of the microbiome and the decreased effectiveness of immunotherapy. Assuming that a prospective clinical trial randomizing based on concomitant medication will not conducted, additional observational studies with alternative designs are needed to resolving this matter, for example a study design including a control group not treated with immunotherapy. If the association is causal one would expect the association to be more pronounced with immunotherapy compared to chemotherapy. The aim of the present study is to test this hypothesis.

## Patients and methods

### Study design

This is a retrospective matched cohort study using clinical data from six hospitals of the Santeon hospital network.

### Data sources

The Santeon hospital network consists of seven large teaching hospitals geographically spread across the Netherlands. The Netherlands Cancer Registry (NCR) was used for identifying all patients diagnosed with NSCLC in a Santeon hospital, and for obtaining information on the date of diagnosis and the vital status. Individual patients are assigned a unique anonymous identifier, which enables them to be tracked in the Santeon Farmadatabase (SFD). The information of the SFD was used for collection of detailed information about the systemic treatments^14^. Finally, the patients’ medical records were used to complement the database with detailed information about the clinical and demographic characteristics, the use of concomitant medication and the treatment response. All data were gathered and stored at a Research Electronic Data Capture database (REDcap)^15^.

### Study population

Patients diagnosed with stage IV NSCLC between 1st January 2015 to 1^st^ January 2019 and who started first-, second- or third-line immunotherapy before the 1^st^ of January 2020 were assigned to the immunotherapy group. We matched every patient in the immunotherapy group to a patient with stage IV NSCLC who received conventional chemotherapy in the pre-immunotherapy era, and has been diagnosed before 1^st^ January 2015 (see publication of Cramer et al. for more details about this cohort)^15^. Patients were (1:1) matched on gender, age groups (< 50 year, 50–60 year, 61–70 year, 71–80 year, > 80 year) and line of treatment (first, second or third).

### Clinical characteristics

The patient characteristics and demographics were collected manually from the patients’ medical records, including age, gender, body mass index (BMI), Eastern Cooperative Oncology Group-Performance status (ECOG-PS), the histology subtype, brain metastases and lines of systemic treatments. First-line treatment (1L) was defined as the initial systemic therapy used in the treatment of NSCLC. Second-line and third-line treatment (2L and 3L) were defined as the therapy given after discontinuation due to disease progression or completion of first or second-line treatment, respectively. Additionally, oncogenic driver mutation status (e.g. EGFR, ALK, ROS-1) and PD-L1 expression were collected if available.

#### Concomitant medication

Patient records were reviewed to collect information on the use of concomitant medication potentially affecting the microbiome^10,11^. Out of the drugs classes known to alter the composition of the microbiome, most commonly used classes in NSCLC patients were selected. The drug classes selected were antibiotics, proton-pump inhibitors, metformin, antidiabetics and opioids. Medication reconciliation for NSCLC patients was performed by the treating oncologist of assisting nurse prior to the start of the systemic treatment and was reported in the medical health record. Exposure was defined if any information on the use of these drugs was reported within a timeframe of 30 days before and 30 days after the start of either immunotherapy or chemotherapy. This timing was chosen because there is evidence that the effects are the strongest when used shortly before and after the start of immunotherapy and consequently, we would expect the largest effect modification by chemotherapy vs immunotherapy (if any)^16^. The 30 days timeframe is also in line with many previous reports on the topic^9,13,17^.

#### Clinical outcomes

OS was defined as the time from the start of systemic therapy to death. Patients still alive at the end of follow-up on 1 January 2020 were censored at this date.

### Statistical analysis

Statistical Software (SPSS version 26 for Windows: IBM) was used for statistical analysis. Categorical and continuous variables were summarized using descriptive statistics. To compare the immunotherapy and chemotherapy group, we used chi-squared tests (categorical variables) and independent t- test (continuous variables). The potential impact of concomitant medication on OS was analyzed through multivariable cox regression analyses. Possible factors associated with OS were first identified using a univariable analysis. All univariate predictors with a *p*-value ≤ 0.15 and three other relevant variables—type of treatment, the ECOG-PS and the use of antibiotics—were used to construct the multivariable model. In the final models, backward selection was applied to eliminate non-significant variables (*p*-value ≤ 0.10). Finally, the models were examined for the existence of effect modification by statistical testing of an interaction term between concomitant drugs of interest and the type of treatment (chemotherapy or immunotherapy). In order to investigate the difference between the lines of treatment, an exploratory analysis was performed for 1L patients and for 2L + 3L patients using the same approach described above. Survival curves using the Kaplan–Meier method were constructed to visualize—where considered of relevance- contrast between the use of concomitant medication and the type of treatment (chemotherapy or immunotherapy).

### Ethical statement

All methods were carried out in accordance with relevant guidelines and regulations. Our ethics committee—the Santeon Institutional Review Board—approved the study (SDB 2019–013) and waived the need for informed consent because of anonymous data handling and the retrospective nature of the study. The study was performed in accordance with the ethical standards of the institutional and national research committee and with the 1964 Helsinki declaration and its later amendments or comparable ethical standards.

## Results

### Baseline characteristics

A total of 221 immunotherapy patients could be matched to patients treated with chemotherapy in the pre-immunotherapy era resulting in a total of 442 patients available for our primary analysis. The baseline characteristics of the immunotherapy and chemotherapy group are summarized in Table 1. Both immuno- and chemotherapy groups had an average age of 64 years. The majority of patients was male (59.2%) and received immunotherapy as second or higher line of treatment (62%). The demographic and baseline characteristics of the patients were well balanced between the two treatment groups, with the exception of the ECOG-PS. In the immunotherapy group, there was a significantly higher proportion of patients with an ECOG -PS1 (62.4% versus 39.8%) as compared to the chemotherapy group. In both groups, the most common histology subtype was adenocarcinoma, although there were fewer patients in the immunotherapy group with squamous tumor histology than in the chemotherapy group (10.9% versus 18.1% respectively). Table 2 provides a list of concomitant medication in use at the time of start systemic treatment. The most commonly used drugs in both groups were PPIs (43.4% vs 45.7% for the immuno- and chemotherapy group). Patients who received immunotherapy used significantly less opioids (26.7% vs 37.6% *p* = 0.02) and used more antibiotics as compared to the patients who received chemotherapy (15.8% vs 19.5%, *p* = 0.32). Only a small percentage of patients in the immunotherapy and chemotherapy group used metformin (3.6% and 9.5% respectively) or other antidiabetics (2.3% and 3.2% respectively).

**Table 1:** Patient characteristics of stage IV NSCLC patients treated with immunotherapy and chemotherapy.

Characteristics	Immunotherapy (n = 221)	Chemotherapy (n = 221)	*p*-value
Age (median ± SD)	64.0 ± 9.0	64.1 ± 8.9	0.83
Gender	131 (59.2%)	131 (59.2%)	1.0
BMI (median ± SD)	25.0 ± 6.3	25.0 ± 4.1	0.82
**Line of treatment**			1.0
1	84 (38.0%)	84 (38.0%)	
2	120 (54.3%)	120 (54.3%)	
3	17 (7.7%)	17 (7.7%)	
**ECOG PS**			< 0.001
0	70 (31.7%)	104 (47.1%)	
1	138 (62.4%)	88 (39.8%)	
2	10 (4.5%)	19 (8.6%)	
≤ 3	2 (0.9%)	4 (1.8%)	
Unknown	1 (0.5%)	6 (2.7%)	
**Immunotherapy**			
Nivolumab	121 (54.8%)		
Pembrolizumab	93 (42.1%)		
Atezolizumab	7 (3.0%)		
Chemotherapy			
Cisplatin/pemetrexed		14 (6.3%)	
Cisplatin/gemcitabine		5 (2.3%)	
Carboplatin/pemetrexed		57 (31.3%)	
Carboplatin/gemcitabine		17 (7.7%)	
Carboplatin/paclitaxel/bevacizumab		21 (9.5%)	
Pemetrexed monotherapy		46 (20.8%)	
Docetaxel monotherapy		58 (26.2%)	
Other		3 (1.4%)	
**Histology**			< 0.001
Adenocarcinoma	176 (79.6%)	132 (59.7%)	
Squamous	24 (10.9%)	40 (18.1%)	
Large cell carcinoma	10 (4.5%)	17 (7.7%)	
Other	11 (4.9%)	32 (14.5%)	
**PD-L1 expression**			
< 1%	34 (15.4%)		
1–50%	28 (12.7%)		
> 50%	100 (45.2%)		
Unknown	60 (27.1%)		
**Targetable mutations**			0.58
EGFR	5 (2.3%)	6 (2.7%)	
ALK	0	1 (0.5%)	
Other	5 (2.3%)	0	
Brain metastasis (yes)	49 (22.2%)	43 (19.5%)	0.48

Abbreviations: ECOG PS, Eastern Cooperative Group performance status; BMI, Body mass index; PD-L1, programmed death ligand-1.

**Table 2:** Concomitant medication exposure according to immunotherapy of chemotherapy treatment.

	Immunotherapy (n = 221)	Chemotherapy (n = 221)	*p*-value
Antibiotics	35 (15.8%)	43 (19.5%)	0.32
Antidiabetics	5 (2.3%)	7 (3.2%)	0.56
Metformin	8 (3.6%)	21 (9.5%)	0.12
PPI	96 (43.4%)	101 (45.7%)	0.63
Opioids	61 (27.6%)	83 (37.6%)	0.02

Abbreviations: PPI, proton pump inhibitor.

### Overall survival outcomes in the total population

The results of the univariable and multivariable analysis for OS are summarized in Table 3. The multivariable model showed that the use of antibiotics and opioids was significantly associated with shorter OS, with a corresponding HR of 1.39 (95%CI 1.06–1.81) and a HR 1.33 (95%CI 1.07–1.66) respectively. Proton pump inhibitors, metformin and other antidiabetics were not associated with OS. Overall, the strongest factor associated with OS was the type of systemic treatment (immunotherapy vs chemotherapy). Other factors identified as independently associated with OS in the multivariable model were type of histology (squamous vs. non-squamous) and line of treatment (≥ 2L vs. 1L).

**Table 3:** Univariable and multivariable model for overall survival in the total population.

	Univariable model			Multivariable model		
	HR	95% CI	p-value	HR	95% CI	*p*-value
**Overall survival for the total population (n = 442)**						
Treatment immunotherapy (vs chemotherapy)	0.45	0.36–0.56	< 0.01	0.46	0.37–0.58	< 0.01
Histology squamous (vs non-squamous)	1.38	1.04–1.84	0.03	1.33	0.10–1.79	0.05
**ECOG PS**						
1 (vs 0) ≥ 2 (vs 0)	1.00 1.40	0.80–1.25 0.95–2.06	0.99 0.09	1.08 1.24	0.86–1.35 0.84–1.83	0.52 0.28
Gender female (vs. male)	0.97	0.78–1.19	0.75			
Age ≥ 75 year ( vs. < 75 year)	0.80	0.55–1.16	0.24			
BMI ≥ 25 (vs. < 25)	0.99	0.76–1.28	0.92			
Brain metastases yes (vs. no)	1.08	0.84–1.39	0.53			
Line of treatment ≥ 2 (vs. 1)	1.45	1.17–1.80	< 0.01	1.44	1.16–1.78	< 0.01
Antibiotics use (vs. no use)	1.32	1.01–1.71	0.04	1.39	1.06–1.81	0.02
Antidiabetics use (vs. no use)	1.57	0.86–2.87	0.14	1.37	0.74–2.52	0.32
Metformin use (vs. no use)	1.33	0.89–1.98	0.17			
PPI use (vs. no use)	1.16	0.94–1.42	0.17			
Opioid use (vs. no use)	1.48	1.19–1.84	< 0.01	1.33	1.07–1.66	0.01

Abbreviations: ECOG PS, Eastern Cooperative Group performance status; BMI, body mass index PPI, proton pump inhibitor; HR, Hazard ratio; CI, confidence interval.

For both antibiotics and opioids, the tests for interaction with treatment type were insignificant, both with a p value of 0.50. The distinctive effects of antibiotics and opioids on OS for the immuno- and chemotherapy cohort were summarized in Table 4a. The calculated point estimates for the hazard ratio for use of antibiotics towards OS were similar with immunotherapy (HR 1.20 95% CI 0.79–1.85) and chemotherapy (HR 1.46 95%CI 1.04–2.05). The same was observed for the use of opioids (HR 1.44 (95%CI 1.01–2.06) in immunotherapy group and a HR of 1.24 (95%CI 0.94–1.63) in chemotherapy group).

**Table 4:** Multivariable model for OS within the total population (a) and the 1L (b) for both antibiotic and opioid exposure according to type of treatment (chemotherapy or immunotherapy).

	HR (95% CI) for antibiotic use (vs no use) according to the type of treatment	*p*-value
**a. OS total population**		
**Antibiotic use**^**a,1**^		
Chemotherapy cohort	1.46 (1.04–2.05)	0.03
Immunotherapy cohort	1.20 (0.79–1.85)	0.39

	HR (95% CI) for opioid use (vs no use) according to the type of treatment	*p*-value
**Opioid use**^**a,2**^		
Chemotherapy cohort	1.24 (0.94–1.63)	0.13
Immunotherapy cohort	1.44 (1.01–2.06)	0.04

	HR (95% CI) for antibiotic use (vs no use) according to the type of treatment	*p*-value
**b. OS first line**		
**Antibiotic use**^**b.3**^		
Chemotherapy cohort	1.54 (0.86–2.76)	0.15
Immunotherapy cohort	1.45 (0.75–2.77)	0.27

	HR (95% CI) for opioid use (vs no use) according to the type of treatment	*p*-value
**Opioid use**^**b.4**^		
Chemotherapy cohort	1.30 (0.80–2.09)	0.29
Immunotherapy cohort	2.27 (1.24–4.16)	< 0.01

The multivariable model includes the variables:

^a^Treatment (immunotherapy vs chemotherapy), histology (squamous vs n*on-squamous), line of treatment* (≥ 2 vs. 1), antibiotic (use vs no us*e)*, opioids (use vs no use).

^1^Interaction between antibiotic use and type of treatment.

^2^Interaction between opioid use and type of treatment.

^b^Treatment (immunotherapy vs chemotherapy), brain metastases (yes vs no), antibiotic (use vs no use),opioids (use vs no use).

^3^Interaction between antibiotic use and type of treatment.

^4^Interaction between opioid use and type of treatment.

### Overall survival outcomes per treatment line

**Figure 1:**
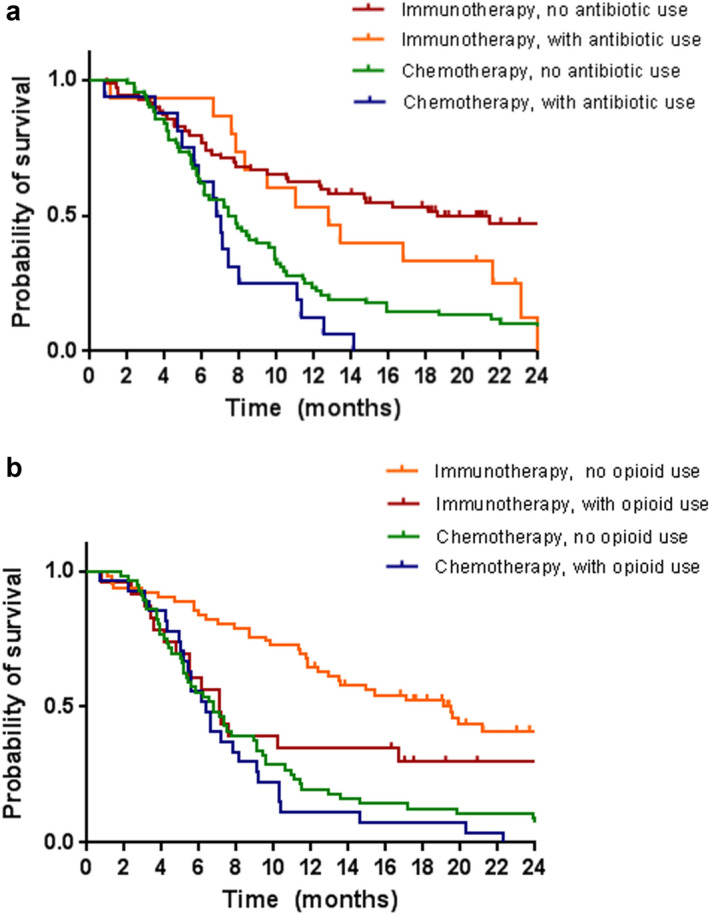
Kaplan–Meier curves showing overall survival for first-line chemo- and immunotherapy with and without the use of antibiotics (**a**) and with and without the use of opioids (**b**).

Restriction of the cohort to patients with 1L treatment yielded similar HRs in that setting. The HRs were 1.49 (95% CI 0.97–2.29) for antibiotic use and 1.58 (95% CI 1.08–2.32) for opioid use (Supplementary Table S1). Also in this setting there was no association between OS and proton pump inhibitors, metformin and other antidiabetics. Factors significantly associated with OS in the multivariable model were the type of treatment (immunotherapy vs chemotherapy) and the presence of brain metastases. The tests for interaction between treatment type and the use of antibiotics and opioids were both not statistically significant, with corresponding p values of 0.89 and 0.15 respectively. The negative effects of antibiotics and opioids on OS were observed in patients treated with chemotherapy as well as in patients treated with immunotherapy (Table 4b), with HRs of similar magnitude between the immuno- and chemotherapy group, although not all were statistically significant. In a second line setting, the use of antibiotics and the use opioids were not statistically significantly associated with OS. The multivariable model for OS showed that only the type of treatment (immunotherapy vs chemotherapy) was associated with a lower risk of death (Supplementary Table S2). Figures 1a and b demonstrate the Kaplan–Meier survival curves for patients treated with immuno- versus chemotherapy, and using/not using antibiotics (1a) or using/not using opioids (1b) in the 1L.

## Discussion

In this historically matched cohort study of 442 stage IV NSCLC patients, the use of antibiotics and opioids were shown to be independently associated with worse survival outcomes after adjusting for other prognostic factors associated with a poorer prognosis. Moreover, the negative influence of antibiotics and opioids on OS was observed both for treatment with chemotherapy as well as with immunotherapy. This finding suggests that the association between worse survival outcomes and the use of antibiotics and opioids more likely originates from its use being linked with confounding factors rather than the disturbing effect these drugs have on the gut microbiome.

We believe that our historically matched cohort study design adds to what has been published about the topic so far because all previously published reports on the association of antibiotics with immunotherapy effectiveness are retrospective single arm cohort studies. Lurienne et al. (2020) summarized these cohort studies (n = 21) in a meta-analysis and reported a pooled HR for OS of 1.69 (95% CI 1.25–2.29) for NSCLC patients exposed to antibiotics when starting immunotherapy^16^. Our observed HR of 1.39 is in line with this pooled number but appears of similar magnitude in patients treated with chemotherapy in the years that immunotherapy was not yet available. This finding argues against causality because an effect of antibiotics on chemotherapy effectiveness as a result of microbiome disturbance is unlikely. Thus, we consider that residual confounding by indication bias plays a role in previous reports. An exception is the recent study of Chalabi et al. In a post hoc analysis of a randomized clinical trial of atezolizumab versus docetaxel in a second line setting, a larger association between antibiotics and worse OS was observed in the atezolizumab study arm compared to chemotherapy^17^. However, also in that study residual confounding cannot be excluded. It is also conceivable that symptomatic rapid progressive disease represented by antibiotic use, prevented patients randomized to immunotherapy from early disease control resulting in earlier death eventually^18^. Finally, Cortellini et al. recently reported a non-significant HR of 1.42 for antibiotics in patients treated with the combination of immunotherapy and chemotherapy in first line. This HR aligns very much with our observed HR in patients receiving chemotherapy (1.54), further supporting the hypothesis that recent antibiotic use is primarily a prognostic factor instead of an etiological factor^19^.

For opioid use our findings align with what has been studied by Zheng et al.^20^. The authors conducted a cohort analysis (n = 203 patients) and a meta-analysis (n = 26 articles) to investigate the impact of opioids use on survival of cancer patients. The results of their analyses showed a negative association between cancer-specific survival and the use of opioids explained cancer-related pain treated with opoids. Furthermore, other studies have reported that opioids may negatively affect cancer patients’ survival through respiratory depression, delirium, addiction or directly by acting on tumor cells^21,21^. Even though these authors did not investigate whether the negative effects of opioids on survival outcomes differ between patients treated with immunotherapy or chemotherapy, it supports our observed association between opioids and survival irrespective of the systemic treatment applied.

Noteworthy is that the use of PPIs, metformin and other antidiabetics were not associated with poor survival outcomes in our cohort. This might indicate that both the microbiome-hypothesis and the hypothesis of confounding by indication should be rejected for these drugs. Interesting, however, is that some other studies observed associations between PPI use and worse outcomes^15^. That we were unable to replicate this might be explained by the very high prevalence of PPI use in our cohort that could have resulted in overshadowing an assumed proxy of PPI use for worse clinical condition. In the Netherlands, PPIs are recommended for every adult > 65 years and using anti-platelet drugs, NSAIDS or corticosteroids. Nevertheless, our data suggest no effect of PPIs on effectiveness of immunotherapy in NSCLC patients. The frequency of patients using metformin (6.5%) and antidiabetics (2.7%) in our cohort we consider too small to conclude on any association between these drugs and survival outcomes.

### Strengths and weaknesses

Our study has a number of notable strengths. The main strength is its matched cohort study design. This design enabled us to investigate the possibility of concomitant medication-use having a prognostic impact on patients’ clinical survival, regardless of the treatment given, under the assumption that unmeasured confounding factors will be balanced between the two groups. That we captured chemotherapy patients from a period that immunotherapy was not yet available maximized the potential for balance because after market access of immunotherapy patient characteristics are more likely to guide the decision for either treatment. Nevertheless, the distribution of the ECOG status appeared imbalanced in our study. The most likely explanation for this is that the milder toxicology from immunotherapy has led to patients who would not qualify for toxic chemotherapy starting with immunotherapy anyway. By fixing the ECOG status in all our multivariable regression models we think that adjustment for this imbalance has been maximized. Another positive aspect is that we were able to evaluate not only the effects of antibiotics on the therapeutic outcomes of NSCLC patients but also the effects of other drugs, known to be associated with changes of the microbiome composition. There are numerous observational studies investigating the impact of antibiotics, however, studies regarding the effects of other drugs are scarce. Lastly, compared to previous reports, we used a relatively large cohort with advanced NSCLC patients treated in six different hospitals across the Netherlands.

Limitation of our study is the small number of patients using some of the drugs under study. This might explain some null findings due to limited statistical power. Another limitation, in hindsight, is that we did not collect data about the use of corticosteroids in our patients. Corticosteroids have been linked to altered immunotherapy effectiveness as well and inclusion of this variable might have resulted in other HRs with our variables of interest^23,24^. Some co-linearity of corticosteroids and antibiotics or PPIs cannot be ruled out. Another potential drawback is that we examined concomitant medication when used within a relative short time frame of 30 days before up to 30 days after the start of the systemic therapy. Although the decision for this time frame was well-thought-out (see Methods), we acknowledge that this focus prevented us from studying time varying associations. Finally, our study shares all limitations linked to retrospective studies, at least information bias. For example, the use of concomitant medication was fully based on the information recorded in the patients’ health records and may not be completely comprehensive. On the other hand, both patients with chemotherapy and with immunotherapy were captured from the same hospitals, making the risk of misclassification bias, if any, being non-differential.

## Conclusion

In conclusion, we observed that the use of antibiotics and opioids is associated with shorter survival similarly in chemotherapy and immunotherapy treated NSCLC patients. This suggests that the association is likely to be a consequence of confounding rather than disturbing the composition of the microbiome.

## Supplementary Information

Supplementary Information.
