# Supplementary material for: The association between gut microbiome affecting concomitant medication and the effectiveness of immunotherapy in patients with stage IV NSCLC
Source: Sci Rep. 2021 Dec 2;11:23331. doi: 10.1038/s41598-021-02598-0 (PMC8640057; doi:10.1038/s41598-021-02598-0)
Supplement: Supplementary file 1 — Supplementary Information. [file 41598_2021_2598_MOESM1_ESM.docx]

**Appendix

The association between gut microbiome affecting concomitant medication and the effectiveness of immunotherapy in patients with stage IV NSCLC**

M.V. Verschueren^1^
C.M. Cramer – van der Welle^2^
M. Tonn^1^
F.M.N.H. Schramel^3^B.J.M. Peters^1^
E.M.W. van de Garde^1,4^

^1^ Department of Clinical Pharmacy, St. Antonius Hospital, Utrecht/Nieuwegein, the Netherlands
^2^ Santeon Hospital Group, Utrecht, The Netherlands ^3^ Department of Pulmonary Diseases, St. Antonius Hospital, Utrecht/Nieuwegein, The Netherlands
^4^ Division of Pharmacoepidemiology and Clinical Pharmacology, Department of Pharmaceutical Sciences, Utrecht University, Utrecht, The Netherlands

Corresponding author: M.V. Verschueren, [m.verschueren@antoniusziekenhuis.nl](mailto:m.verschueren@antoniusziekenhuis.nl), +31641327430. St Antonius hospital, Koekoekslaan 1, 3435 CM, Nieuwegein, the Netherlands.

Abstract word count: 242 words
Manuscript word count: 2994 words

Table S1. Univariable and multivariable model for overall survival in first-line treatment

| **Overall survival for first-line treatment (n = 168)** | | | | | | |
| --- | --- | --- | --- | --- | --- | --- |
|  | **Univariable model** | | | **Multivariable model** | | |
|  | HR | 95% CI | p-value | HR | 95% CI | p-value |
| Treatment immunotherapy (vs chemotherapy) | 0.37 | 0.26 - 0.54 | <0.01 | 0.37 | 0.26 - 0.54 | <0.001 |
| Histology squamous (vs non-squamous) | 1.29 | 0.80 - 2.08 | 0.30 |  |  |  |
| ECOG PS |  |  |  |  |  |  |
| 1 (vs 0) ≥ 2 (vs 0) | 1.06 1.59 | 0.73 – 1.53 0.86 – 2.93 | 0.76 0.17 | 1.02 1.55 | 0.70 – 1.48 0.83 – 2.90 | 0.92 0.17 |
| Gender female (vs. male) | 1.10 | 0.78 - 1.56 | 0.60 |  |  |  |
| Age ≥75 year ( vs. <75 year) | 0.85 | 0.51 - 1.41 | 0.85 |  |  |  |
| BMI ≥ 25 (vs. <25) | 1.09 | 0.72 - 1.65 | 0.68 |  |  |  |
| Brain metastases yes (vs. no) | 1.46 | 0.99 - 2.14 | 0.06 | 1.54 | 1.04 - 2.28 | 0.03 |
| Antibiotics use (vs. no use) | 1.46 | 0.96 - 2.21 | 0.08 | 1.49 | 0.97 - 2.29 | 0.07 |
| Antidiabetics use (vs. no use) | 2.14 | 0.53 - 8.70 | 0.29 |  |  |  |
| Metformin use (vs. no use) | 1.77 | 0.10 - 3.15 | 0.05 | 1.07 | 0.58 - 1.97 | 0.83 |
| PPI use (vs. no use) | 1.24 | 0.87 - 1.75 | 0.24 |  |  |  |
| Opioids use (vs. no use) | 1.75 | 1.20 - 2.54 | <0.01 | 1.58 | 1.08 - 2.32 | 0.02 |

Abbreviations: ECOG PS, Eastern Cooperative Group performance status; BMI, body mass index PPI, proton pump inhibitor; pump inhibitor; HR, Hazard ratio; CI, confidence interval.

Table S2. Univariable and multivariable model for overall survival in second- and third line treatment

| **Overall survival for second- and third line treatment (n = 274)** | | | | | | |
| --- | --- | --- | --- | --- | --- | --- |
|  | **Univariable model** | | | **Multivariable model** | | |
|  | HR | 95% CI | p-value | HR | 95% CI | p-value |
| Treatment immunotherapy (vs chemotherapy) | 0.51 | 0.39 - 0.66 | <0.01 | 0.52 | 0.40 - 0.68 | <0.01 |
| Histology squamous (vs non-squamous) | 1.47 | 1.03 - 2.10 | 0.03 | 1.34 | 0.93 - 1.94 | 0.12 |
| ECOG PS |  |  |  |  |  |  |
| 1 (vs 0) ≥2 (vs 0) | 0.98 1.31 | 0.74 – 1.29 0.80 – 2.15 | 0.87 0.29 | 1.10 1.11 | 0.82 – 1.48 0.66 – 1.86 | 0.51 0.69 |
| Gender female (vs. male) | 0.91 | 0.70 - 1.18 | 0.47 |  |  |  |
| Age ≥75 year ( vs. <75 year) | 0.89 | 0.51 - 1.56 | 0.68 |  |  |  |
| BMI ≥ 25 (vs. <25) | 0.85 | 0.60 - 1.19 | 0.34 |  |  |  |
| Brain metastases yes (vs. no) | 0.89 | 0.64 - 1.25 | 0.52 |  |  |  |
| Antibiotics use (vs. no use) | 1.28 | 0.91 - 1.80 | 0.16 | 1.30 | 0.92 - 1.84 | 0.14 |
| Antidiabetics use (vs. no use) | 1.29 | 0.66 - 2.52 | 0.46 |  |  |  |
| Metformin use (vs. no use) | 1.11 | 0.63 - 1.94 | 0.73 |  |  |  |
| PPI use (vs. no use) | 1.04 | 0.81 - 1.35 | 0.75 |  |  |  |
| Opioids use (vs. no use) | 1.31 | 1.00 - 1.71 | 0.05 | 1.23 | 0.94 - 1.61 | 0.14 |

Abbreviations: ECOG PS, Eastern Cooperative Group performance status; BMI, body mass index PPI, proton pump inhibitor; pump inhibitor; HR, Hazard ratio; CI, confidence interval
